# High-resolution CT features of COVID-19 pneumonia in confirmed cases

**DOI:** 10.1186/s43055-020-00236-9

**Published:** 2020-07-07

**Authors:** Suzan Omar, Abdelghany Mohammed Motawea, Rabab Yasin

**Affiliations:** Radiology Department, Faculty of Medicine, Menofia University, Menofia, Egypt

**Keywords:** COVID-19, Coronavirus infections, CT, Computed tomography, X-ray, Pneumonia, Viral, Lung diseases

## Abstract

**Background:**

Coronavirus (COVID-19) pneumonia emerged in Wuhan, China, in December 2019. It was highly contagious spreading all over the world, with a rapid increase in the number of deaths. COVID-19 is characterized by fever, fatigue, dry cough, and dyspnea with variable chest imaging features which have been detected.

In our study, we shared our experience of CT findings in proven cases of COVID-19 to recognize the different CT patterns to help in proper and accurate diagnosis.

**Results:**

The most common CT features detected in COVID-19 cases were ground glass patches (93.3%) followed by subpleural linear abnormality (53.3%), air bronchogram (23.3%), and consolidation patches (23.3%), as well as bronchial wall thickening (16.7%), crazy paving pattern (13.3%), and discrete nodules surrounded by ground glass appearance (10%). Only one case had pleural effusion (3.3%). No cavitary lesions or specific lymph nodes were detected in any of the examined patients. The lung lesions showed typical diffuse, basal, and subpleural involvement with less affection of the upper lobes.

**Conclusion:**

CT imaging findings of COVID-19 can help in early and accurate diagnosis of COVID-19 and proper assessment of the severity of the disease.

## Background

On December 31, 2019, the World Health Organization (WHO) has reported many cases with pneumonia of unidentified cause in Wuhan, China [1].

The first four cases reported are related to the Hunan Seafood Wholesale Market in Southern China, and they were identified by local hospitals using monitoring measures for “pneumonia of unknown origin” that was found after the outbreak of severe acute respiratory syndrome (SARS) in 2003 [2].

On January 7, 2020, coronavirus disease 2019 (COVID-19, or severe acute respiratory syndrome coronavirus 2 (SARS-CoV-2)) was confirmed as the cause of these reported cases, previously known as 2019 novel coronavirus (2019-nCoV), and the outbreak was subsequently named COVID-19 [3].

The WHO announced the outbreak of a worldwide health emergency on January 30, 2020. On February 20, 2020, a total of 75,761 cases and 2130 deaths had been reported in more than 30 countries [4].

The respiratory system is primarily affected by the COVID-19, so the chest imaging is strongly recommended in the suspected cases for both initial evaluation and follow-up [5].

Chest radiographs are of little value in the diagnosis of early stages, but the CT findings may be present early even before the onset of the symptoms. Chest radiographs may be helpful in the intermediate to advanced stages of COVID-19 with features of acute respiratory distress syndrome (ARDS) [6, 7].

Other viral pneumonias may overlap with COVID-19 as regards the chest imaging findings. The findings like ground glass opacities are detected in chest CT with more sensitivity than chest radiographs [8].

Screening test, reverse transcription polymerase chain reaction (RT-PCR), may be initially false negative in the presence of the diagnostic CT findings [8, 9].

With the COVID-19 outbreak and the upgrowing worldwide pandemic with the catastrophic mortality, the inclusive understanding and characterization of the diagnostic imaging findings, the variable criteria, and the chest imaging lineaments are crucial for proper patient management and treatment [10].

In our study, we shared our experience of CT findings in proven cases of COVID-19 to recognize the different CT patterns to help in proper and accurate diagnosis.

## Methods

This was a retrospective study performed during March 2020. High-resolution CT examination of the chest was done for 30 patients who were tested positive for novel coronavirus by nasopharyngeal swap. Age of the patients ranged from 26 to 63 years old with a mean age of 43.56 years. There were 19 males and 11 females with a male to female distribution of 1.7:1.

### High-resolution chest protocol

All images were obtained with the patients in the supine position. Scans were done from the level of the upper thoracic inlet to the inferior level of the costophrenic angle, and the following parameters were used: tube voltage 120 kVp, mAs 250, detector collimation width 2 × 0.25, rotation time 1.0 s, and slice thickness 2.5 mm.

All images are then transmitted to the workstation and picture archiving and communication systems (PACS) for multiplanar reconstruction.

### Image analysis

Imaging findings were analyzed by 3 radiologists with experience of CT chest ranging from 15 to 20 years. The lesions were classified as regards the lobar involvement (single or multiple lobes affected), zonal distribution (upper, basal, or diffuse zonal involvement), and subpleural distribution.

The CT patterns of the lesions were classified into ground glass opacities, crazy paving, or consolidations. The number and shape of patches were described as patchy, large block, or nodular as well as the presence of cavitation.

The interstitial lung involvement was assessed for the presence of reticulations, thickened septa, and subpleural lines. Also, the scans were assessed for bronchial wall thickening, air bronchogram, and pleural effusion of any mediastinal or hilar lymph nodes with specific features.

Each of the five lung lobes was assessed for the degree of involvement and classified as none (0%), minimal (1–25%), mild (26–50%), moderate (51–75%), or severe (76–100%).

No involvement corresponded to a lobe score of 0, minimal involvement to a lobe score of 1, mild involvement to a lobe score of 2, moderate involvement to a lobe score of 3, and severe involvement to a lobe score of 4. An overall lung “total severity score” was reached by summing the five lobe scores (total severity scores ranged from 0–20) (22, 31).

## Results

This was a retrospective study with high-resolution CT examination of the chest which was done for 30 patients who were tested positive for coronavirus by nasopharyngeal swap. Age of the patients ranged from 26 to 63 years old with a mean age of 43.56 years. There were 19 males and 11 females with a male to female distribution of 1.7:1. Average day from the laboratory diagnosis to the clinical symptoms which warranted CT examination was 5.1 days (Table 1).

**Table 1:** Demographic presentations of the cases

Total number of patients	30
**Mean age**	43.56 years
**M/F**	1.7/1
**Average days from diagnosis to CT examination**	5.1 days

The number of the involved lung lobes was assessed (Table 2). There were two patients (6.7%) who had one affected lobe, six patients (20%) who had two affected lobes, nine patients (30%) who had three affected lobes, eight patients (26.7%) who had 4 affected lobes, and five patients (16.6%) who had five affected lobes. According to the degree of involvement of each lobe, the total severity score was calculated (Table 3).

**Table 2:** The number of the affected lobes with total severity score

Number of affected lobes	*N* (%)
**1 affected lobe**	2 (6.7 %)
**2 affected lobes**	6 (20 %)
**3 affected lobes**	9 (30 %)
**4 affected lobes**	8 (26.7 %)
**5 affected lobes**	5 (16.6 %)

**Table 3:** The total severity score

Total severity score	*N* (%)
1–5	4 (13.3%)
6–10	20 (66.7%)
11–15	6 (20%)
16–20	0

The zonal predominance of the lesions was classified as upper, basal, diffuse, and subpleural involvement. Most of the cases show diffuse lung distribution which was seen in 13 patients (43.3 %), basal predominance was seen in 11 patients (36.7 %), and only 6 patients (20%) had upper lobe predominance. Subpleural predominant distribution of the lesions was the common finding seen in 22 patients (73.3%) (Table 4).

**Table 4:** Zonal distributions of the lung lesions

Zonal distribution	*N* (%)
Upper	6 (20%)
Basal	11 (36.7%)
Diffuse	13 (43.3%)
Subpleural distribution	22 (73.3%)

**Fig. 1:**
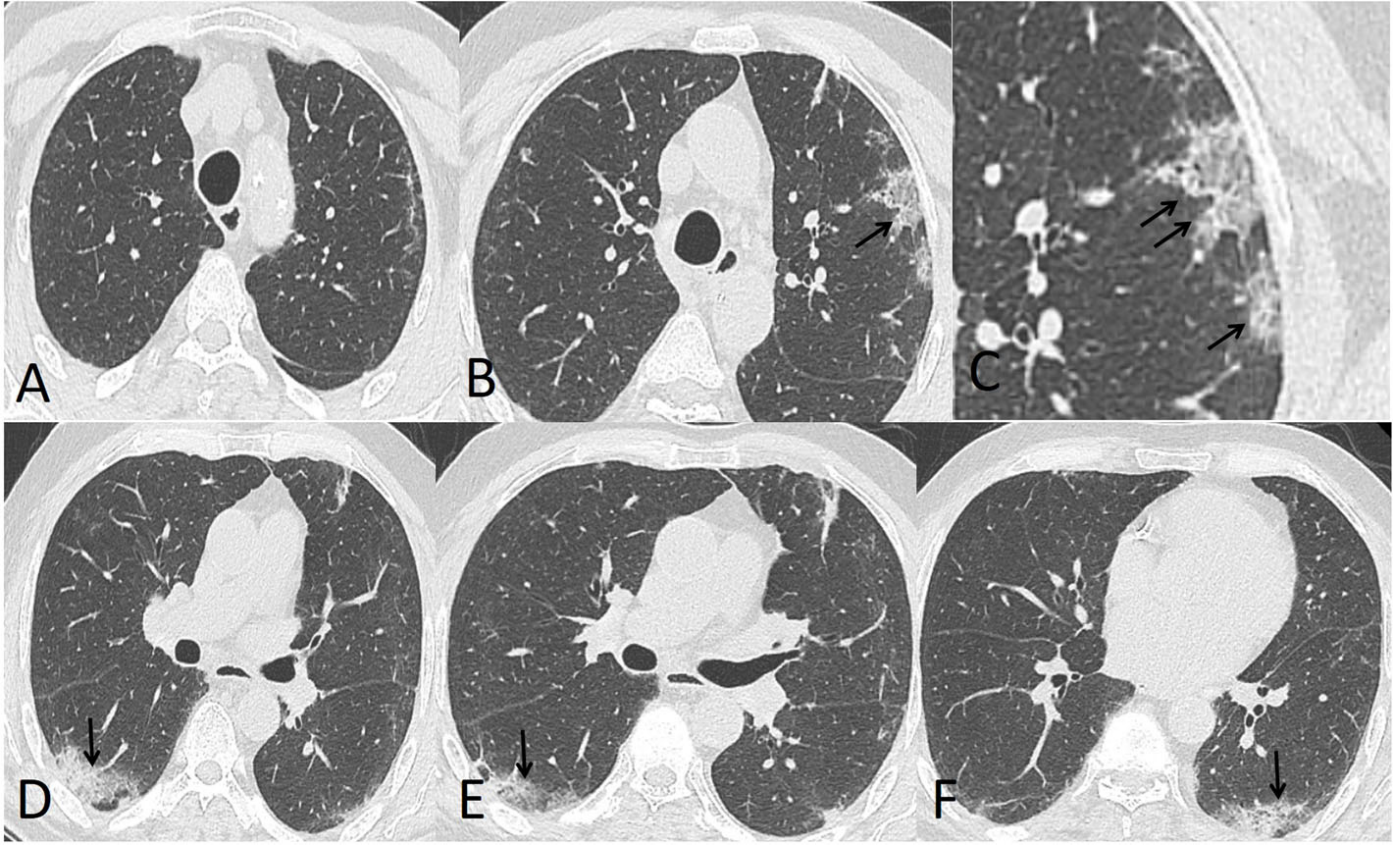
A 39-year-old male. CT chest shows moderate involvement of the left upper lung lobe with subpleural lines (**a**) and crazy paving appearance (arrow in **b**) magnified (arrow in **c**). Bilateral mild lower lobe consolidation patches (arrows in **d**–**f**). Total severity score = 7

**Fig. 2:**
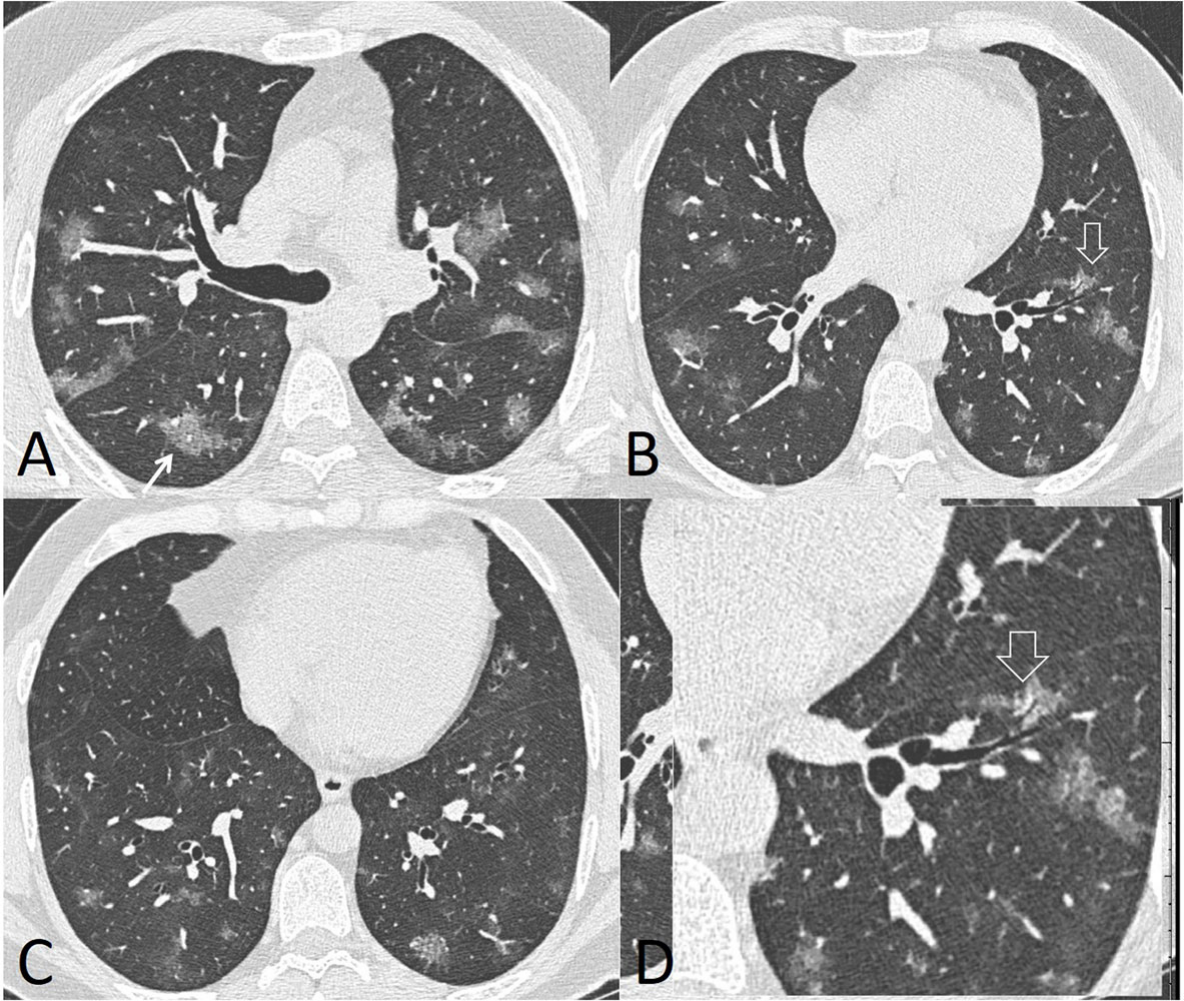
A 46-year-old male patient with bilateral multiple ground glass opacities (white arrow in **a**) and air bronchogram (hollow arrows in **b** and **c**). Total severity score = 8

The patterns of detected CT findings are described in Table 5. The most common CT features detected in COVID-19 cases were ground glass patches (93.3%) followed by subpleural linear abnormality (53.3%), air bronchogram (23.3%), and consolidation patches (23.3%), as well as bronchial wall thickening (16.7%), crazy paving pattern (13.3%), and discrete nodules surrounded by ground glass appearance (10%) (Figs. 1 and 2).

**Table 5:** Different CT patterns of COVID-19

CT pattern	Number of cases (%)
Ground glass opacities (GGO)	28 (93.3%)
Crazy paving patches	4 (13.3%)
Consolidation patches	7 (23.3%)
Lung nodules	3 (10%)
Subpleural fibrotic lines	16 (53.3%)
Bronchial wall thickening	5 (16.7%)
Air bronchogram	7 (23.3%)
Pleural effusion	1 (3.3%)
Mediastinal or hilar lymph nodes	0

**Fig. 3:**
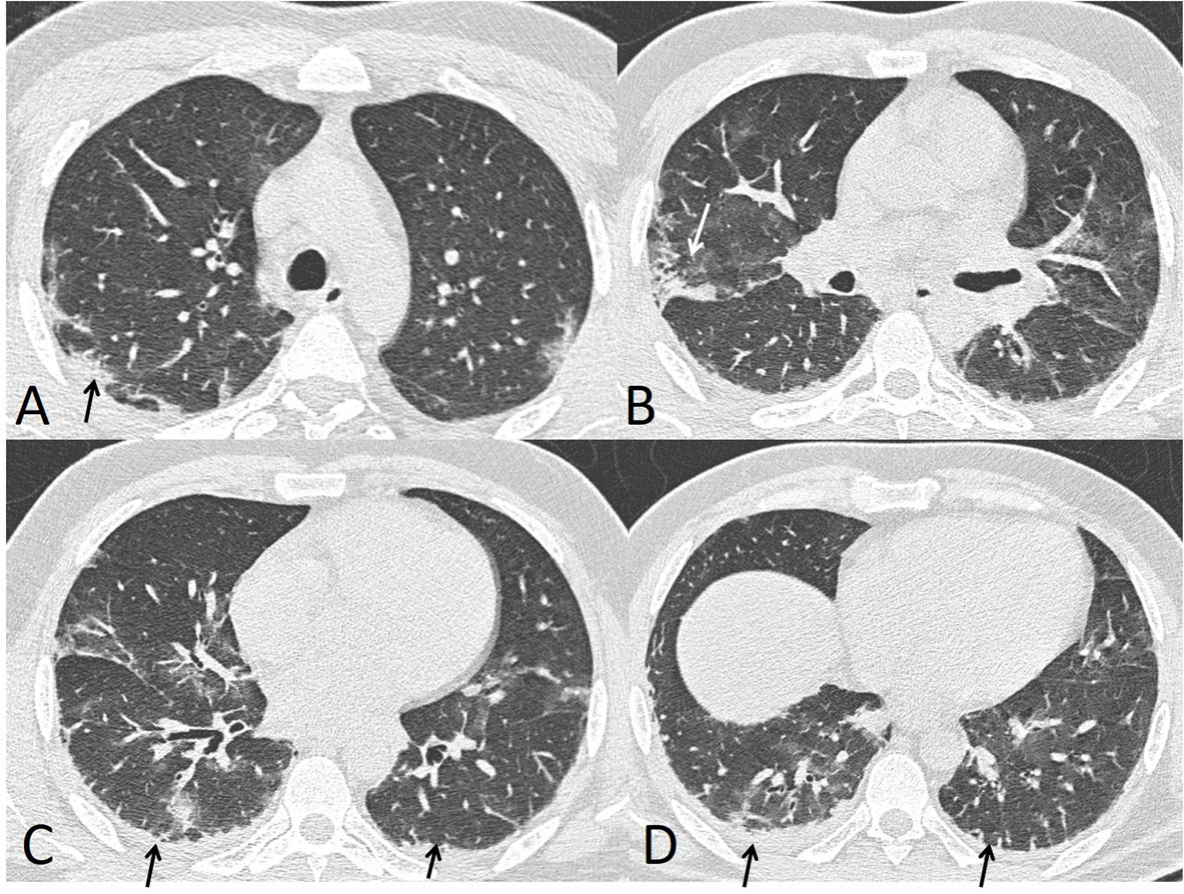
A 48-year-old male with multiple subpleural lines (black arrow in **a**), consolidation patch with air bronchogram (white arrow in **b**), and bilateral minimal pleural effusion (black arrow in **c** and **d**). Total severity score = 12

Only one case had pleural effusion (3.3%) (Fig. 3). No cavitary lesions or specific lymph nodes were detected in any of the examined patients.

## Discussion

In March 2020, coronavirus disease 2019 (COVID-19), caused by severe acute respiratory syndrome coronavirus 2 (SARS-CoV-2), became a worldwide pandemic [11]. Old age and chronic diseases like diabetes and hypertension are associated with high mortality rate of COVID-19 pneumonia, which has sustainable rapid spread in the community [12].

Viral pneumonias notably have a wide variety of imaging features and presentations; some of these features are uncommon or rare in COVID-19 pneumonia such as tree-in-bud opacities and other small nodules, bronchial wall thickening, and bronchial mucus plugs. So, the term “viral pneumonia” has a range of imaging criteria not all of which are typical for COVID19. Also, other lung abnormalities that may be associated with the increased morbidity of COVID-19 pneumonia such as emphysema and diffuse parenchymal lung disease should be described and reported [13].

“COVID-19” CT imaging features are complex due to its significant overlap with other causes of acute lung illness and organizing pneumonia, but recent publications have described COVID-19 imaging features in CT scans; the changes of these features over time can be used for distinguishing COVID-19 from other viral infections [14, 15].

Chest radiographs are the first imaging modality for acute respiratory illness evaluation in immunocompetent patients. Despite chest radiographs’ evident abnormalities of COVID-19, radiographs were normal in about two thirds of the patients. Many studies cited that the CT positive features like ground glass opacities are not evident in the chest radiographs of the same patients [16].

Computed tomography has a main role in the diagnosis and management of COVID-19 respiratory illness [17]. Screening by computed tomography (CT) for COVID-19 identification is not recommended by most radiology professional organizations and societies; however, in persons under investigation (PUI) for COVID-19, the number of CTs performed may increase [18].

**Fig. 4:**
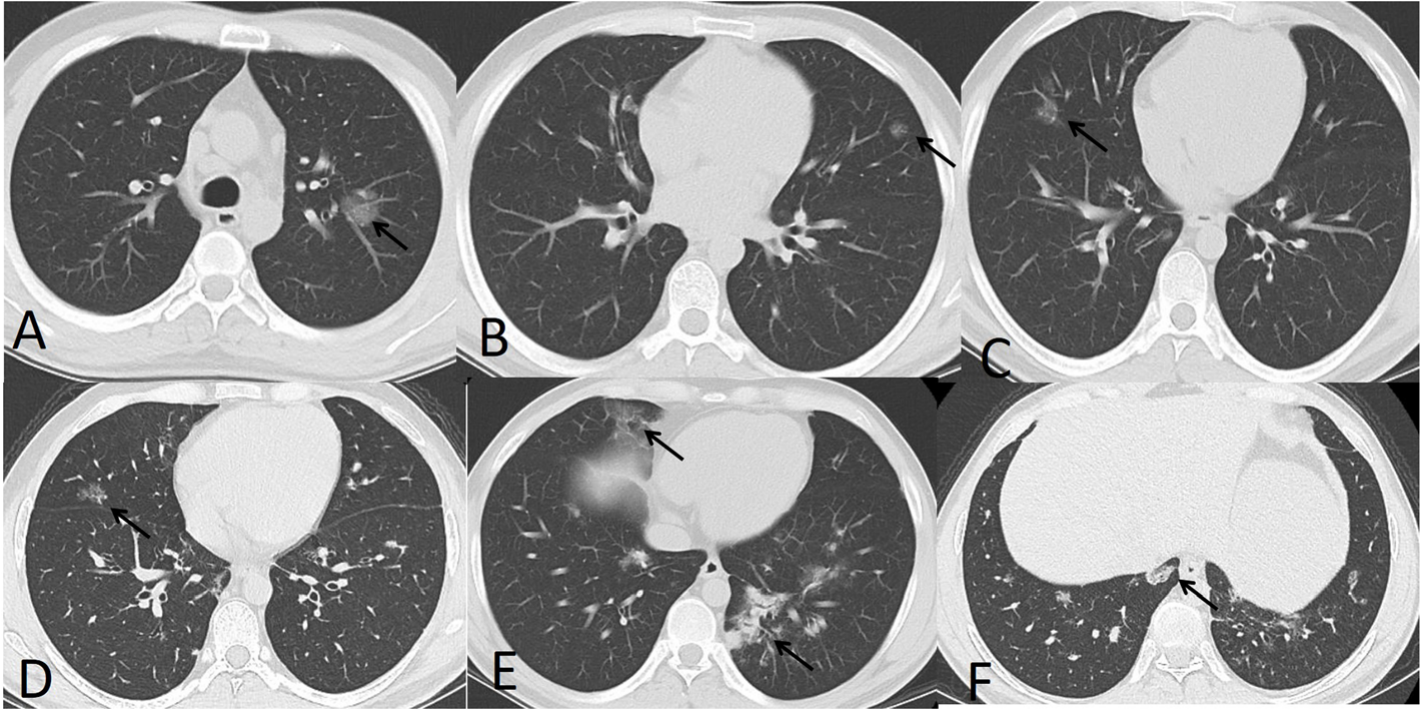
A 34-year-old male presented with multiple rounded ground glass opacities (black arrows in **a**–**d** and **f**) and consolidation patches with air bronchogram (black arrows in **e**). Total severity score = 8

The rates of imaging features also depend on the timing of the imaging in relation to the illness progression. Many cases showed negative CT during the first 2 days after symptom onset with usual development of ground glass opacities within 4 days after symptom onset with a peak at about 6–13 days [19] (Fig. 4). So, the negative chest CT could not be used to exclude the evidence of COVID-19, especially early in the disease. With the progression of the disease, the incidence rates of consolidation patches increase [20].

Jin et al. classified CT findings of COVID-19 to five temporal stages as ultra-early, early, rapid progression, consolidation, and dissipation stages. In 1–2 weeks after exposure (ultra-early stage) which is usually asymptomatic, CT characteristic may show single or multiple focal GGO, patchy consolidative opacities, pulmonary nodules encircled by GGO, and air bronchograms. In the early stage (early symptomatic presentation, 54% of their cases), CT findings include single or multiple GGOs, or GGO combined with interlobular septal thickening. In the rapid progression stage (days 3–7 of symptomatic presentation), CT findings include large, light consolidative opacities and air bronchograms. During the consolidation stage (second week of symptomatic presentation), reductions in density and size of the consolidative opacities may be seen. About 2–3 weeks after the onset, CT may show dispersed patchy consolidative opacities, reticular opacities (referred to as “strip-like opacities”), bronchial wall thickening, and interlobular septal thickening [5].

**Fig. 5:**
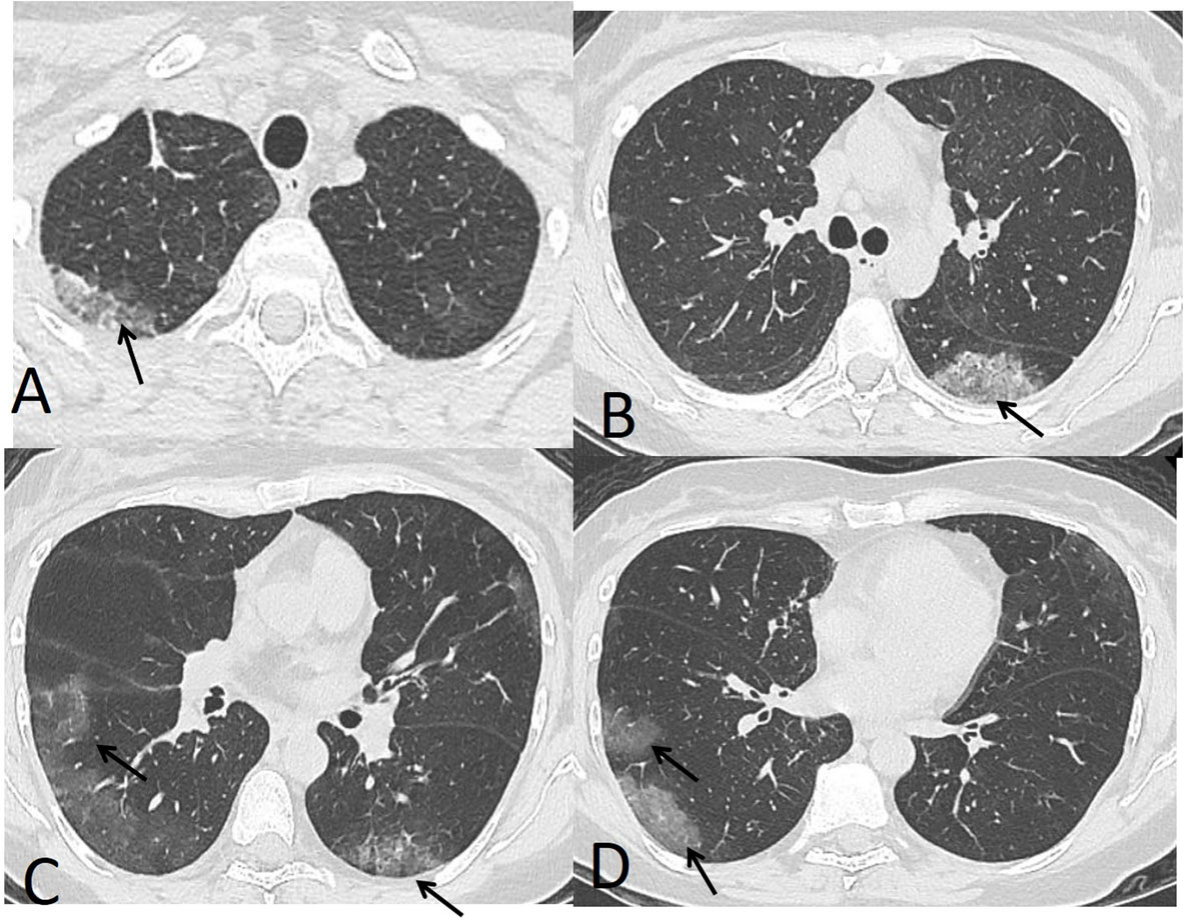
A 44-year-old male with mild involvement of the right upper lobe, consolidation patch (black arrow **a**), and moderate involvement of both lower lobes with multiple subpleural ground glass patchy opacities (arrows on **b**–**d**). Total severity score = 8

**Fig. 6:**
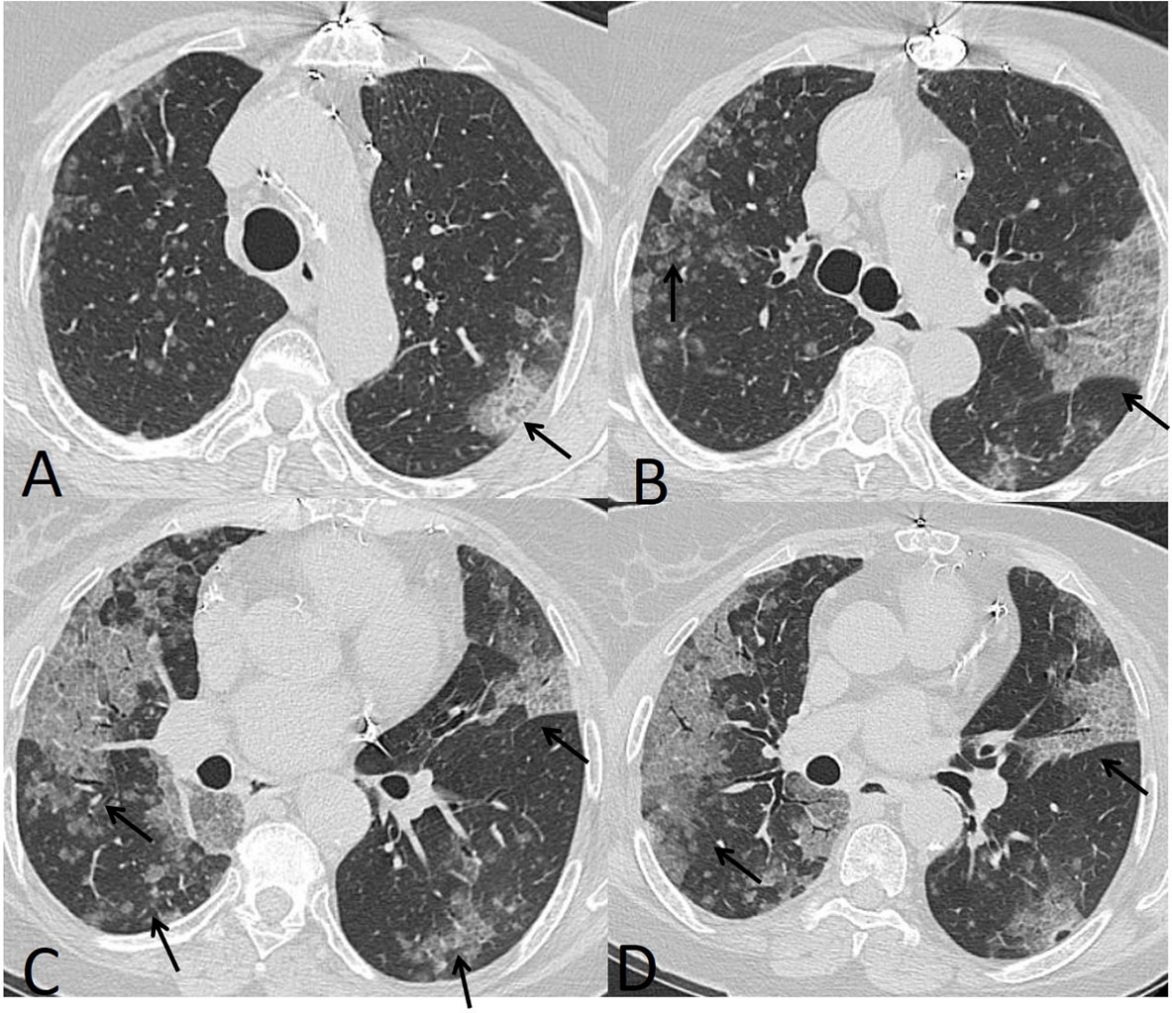
A 57-year-old male with mild involvement of the right upper lobe with multiple rounded nodules of GGO (black arrow **b**), moderate involvement of the left upper lobe with patchy crazy paving (black arrows in **a** and **b**), and severe involvement of the right middle lobe and both lower lobes with patchy crazy paving and air bronchogram (black arrows in **c** and **d**). Total severity score = 15

**Fig. 7:**
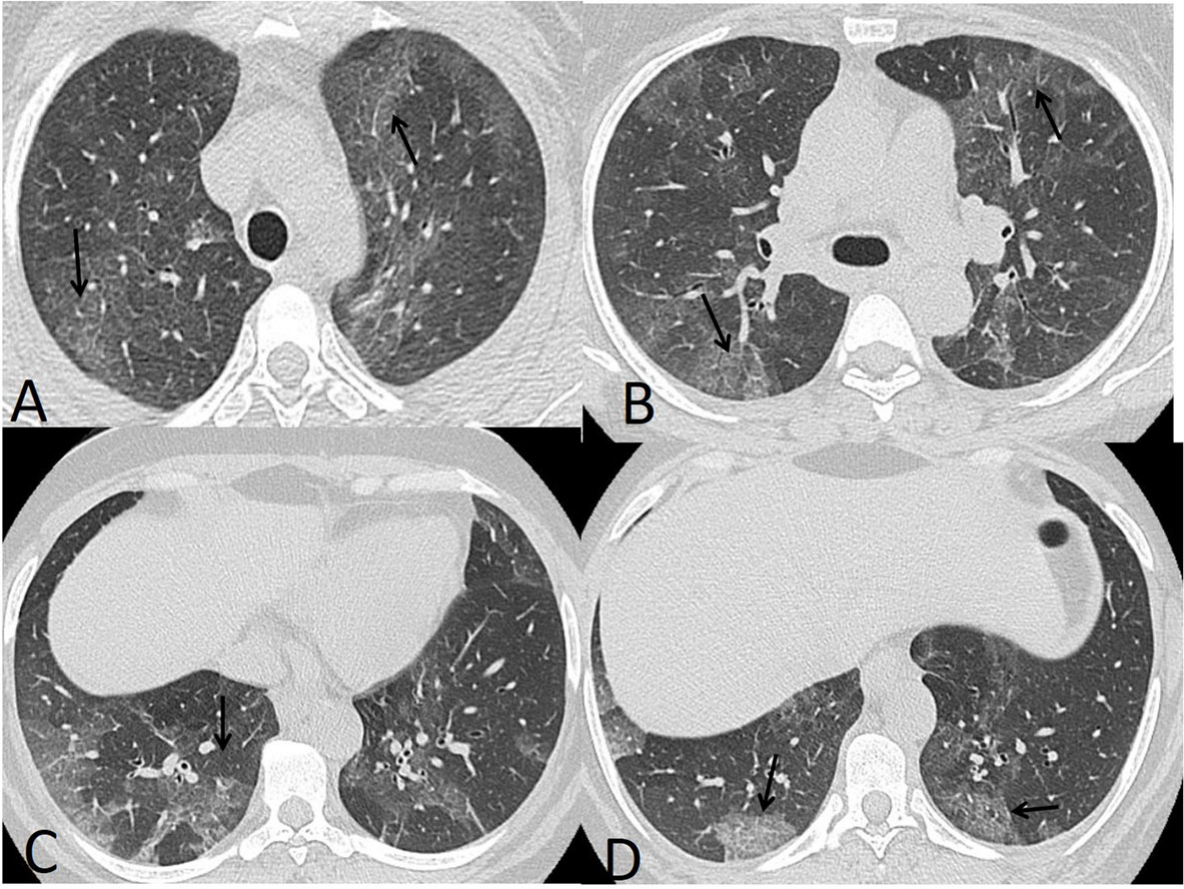
A 53-year-old female with mild involvement of the both upper lobes (arrows in **a** and **b**) and middle lobe (not shown) and moderate involvement of both lower lobes with peripheral subpleural patchy ground glass opacities (arrows in **c** and **d**). Total severity score = 12

In our study, diffuse bilateral lung distribution was the most common feature (43.3 %) with basal prominence (36.7 %) and subpleural involvement (73.3%) (Figs. 5, 6, and 7). Predominant upper lobe affection was uncommon (20%), seen only in 6 patients (Table 4).

**Fig. 8:**
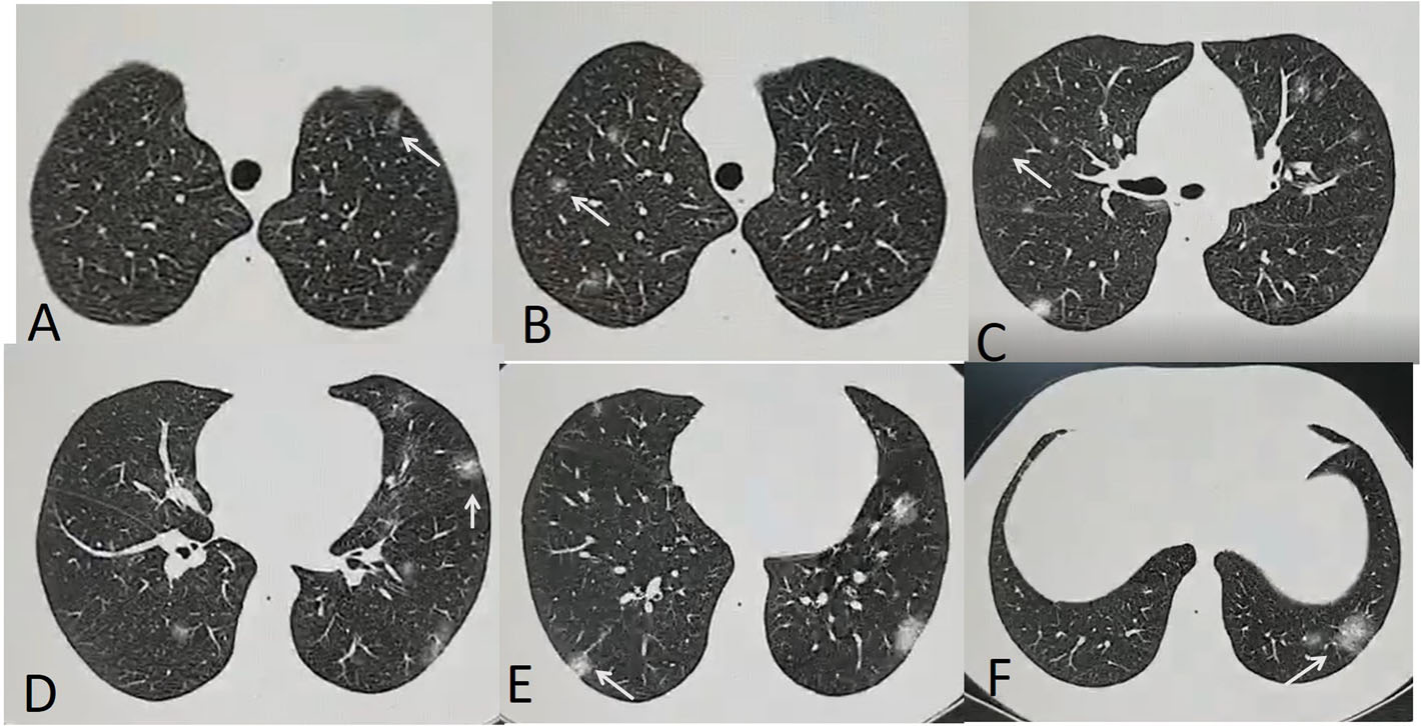
A 32-year-old male with mild involvement of 5 lobes. Multiple nodular opacities with ground glass halo (white arrows). Total severity score = 10

In our study, the most common CT features detected in COVID-19 cases were ground glass patches (93.3%) (Fig. 2), followed by subpleural linear abnormality (53.3%), air bronchogram (23.3%), and consolidation patches (23.3%) (Figs. 1 and 3), as well as bronchial wall thickening (16.7%), crazy paving pattern (13.3%), and discrete nodules surrounded by ground glass appearance (10%) (Figs. 4, 6, and 8).

This agreed with Wang et al. who described ground glass opacities alone or ground glass opacities with consolidative opacities are the most common CT findings. Bilateral involvement with subpleural distribution and absence of pleural effusion are the most common CT features of COVID-19 pneumonia [21].

Also, other studies done on patients of COVID-19 described ground glass opacities with or without consolidation typically in a peripheral, posterior, and diffuse or lower lung zones. Ground glass opacities may have round morphology or a “crazy paving” pattern [22, 23].

These studies have cited that COVID-19 often produces a CT pattern like that of organizing pneumonia, mainly the peripheral ground glass opacities which may be nodular or mass-like, and those are often multilobar and bilateral [24].

Other imaging findings like linear, curvilinear, or perilobular opacities; consolidation; and diffuse ground glass opacities have also been reported; these findings may mimic many diseases like other infections, drug toxicities, and inhalational exposures [25].

Kim et al. described CT features like interlobular septal thickening, pleural thickening, and subpleural involvement with bronchiectasis [6].

In our study, there was only one case with pleural effusion (3.3%). No cavitary lesions or specific lymph nodes were detected in any of the examined patients.

This agreed with other studies which described cavitation, CT halo sign, lymphadenopathy, pleural/pericardial effusion, and pneumothorax as the uncommon CT findings in COVID-19 patients [26].

In other studies, they suggested that the age of the patients would affect the pattern of CT manifestations of COVID-19 as older patients have predominance of consolidative opacities, while younger patients have more GGO [27].

In prospective case studies of 41 diagnosed patients, CT radiological features suggestive of pneumonia were reported in 100% of the patients, of which 98% of cases showed bilateral lung involvement. Lobar and subsegmental consolidations were the common CT findings in the patients admitted to the ICU [28].

In another study of 51 confirmed COVID-19 patients, 1324 lung lesions were detected on chest CT within 1 to 14 days (median, 4 days) of symptom onset. Isolated ground glass opacity, ground glass opacity with reticular or interlobular septal thickening, and ground glass opacity with consolidation patches are the most common CT findings in these patients, respectively. In 80% of these patients, air bronchograms were reported. Multilobular involvement, especially the lower lobes with a peripheral or posterior distribution (or both), was reported in most of the cases [19].

In another study, Chung et al. described the CT findings of COVID-19 pneumonia in 21 patients and reported bilateral lung involvement in 76% and peripheral airspace opacities in 33% of the cases at presentation. Ground glass opacities without consolidative opacities were present in more than 50% of patients, and about 29% showed ground glass opacities with consolidation patches at presentation. The study described the pattern of the lesions as round in 33%, more linear in 14%, and crazy paving pattern in 19%. In 38% of the patients, all lung lobes were affected. The most involvement was in the right lower lobe (76%), and the least common involvement was in the right middle lobe (57%) [22].

Other two large studies with 138 and 99 confirmed cases showed that consolidative airspace opacities (labeled as “patchy shadows”) and ground glass opacities are the most common imaging findings [29, 30].

Also Pan et al. described consistent findings; they reported patchy or punctate GGO in 85.7% and consolidative opacities in 19.0% of 63 patients. In their study, multilobar involvement was reported in about 70% of patients. Other less common findings like interstitial septal thickening (described as “enlarged fibrous stripes”) and pulmonary nodules were also reported [7].

Jin et al. also described typical and atypical chest CT patterns. They reported bilateral, fuzzy edged ground glass opacities with high density and small honeycomb interlobular septal thickening as typical findings in 54.2% of the patients, multiple patchy consolidative opacities were also seen in 31.3% of the patients, and they also found atypical findings such as bronchial wall thickening, pleural effusions, lymphadenopathy, and pulmonary nodules surrounded by ground glass opacities in about 7% of the patients [5].

A significant portion of cases were reported with opacities with unclear or nonspecific distribution. A predominant perihilar pattern was not reported. Bronchial wall thickening, mucoid impactions, and nodules (“tree-in-bud” and centrilobular) seen commonly in infections are not typically reported. Also, lymphadenopathy and pleural effusion have been rarely reported [20].

In our study, there were 4 cases with total severity score 1–5, 20 cases of total severity score 6–10, 6 cases of total severity score 11–15, and no cases in between 16 and 20 total severity score (Table 3).

Chung et al. did a study on 21 cases of COVID-19 and found the total lung severity score ranged from 0 (in the three normal CT examinations) to a maximum of 19, with a mean score of 9.9. The patient with the highest lung severity score was admitted to the intensive care unit [22].

Li et al. assessed the total severity score of COVID-19 patients; they found that TSS for diagnosing severe-critical type was 0.918. The TSS cutoff of 7.5 had 82.6% sensitivity and 100% specificity [31].

The limitations of our study were the small sample size, lack of pediatric cases, and lack of follow-up as well as no lung tissue biopsies were done for histopathologic correlation. There were a limited number of studies done on COVID-19; furthermore, our study was limited to the articles published in English.

We recommend follow-up CT for the patients with prior infection to monitor pulmonary squeal of the disease.

## Conclusion

CT imaging findings of COVID-19 can help in early and accurate diagnosis of COVID-19 and proper assessment of the severity of the disease.

## Data Availability

All data and material are available.

## Abbreviations

COVID-19: Coronavirus disease 2019
CT: Computed tomography
SARS-CoV-2: Severe acute respiratory syndrome coronavirus 2
WHO: World Health Organization
ARDS: Acute respiratory distress syndrome
RT-PCR: Reverse transcription polymerase chain reaction
PACS: Picture archiving and communication systems
GGO: Ground glass opacity
PUI: Persons under investigation
TSS: Total severity score
